# Individualized approach to elexacaftor/tezacaftor/ivacaftor dosing in cystic fibrosis, in response to self-reported anxiety and neurocognitive adverse events: A case series

**DOI:** 10.3389/fphar.2023.1156621

**Published:** 2023-04-27

**Authors:** Hisham Ibrahim, Hammad Danish, David Morrissey, Kevin F. Deasy, Mairead McCarthy, James Dorgan, Claire Fleming, Ciara Howlett, Sarah Twohig, Tamara Vagg, Desmond M. Murphy, Michael Maher, Barry J. Plant

**Affiliations:** ^1^ Cork Centre for Cystic Fibrosis (3CF), Cork University Hospital, University College Cork, Cork, Ireland; ^2^ HRB Clinical Research Facility, University College Cork, Cork, Ireland

**Keywords:** case report, cystic fibrosis transmembrane conductance regulator, CFTR, anxiety, FEV-1, sweat chloride, elexacaftor/tezacaftor/ivacaftor, side-effects

## Abstract

The prevalence of mental health disorders is high among people with Cystic Fibrosis. The psychological symptoms in CF are associated with poor adherence, worse treatment outcomes, and greater health utilization/cost. Mental health and neurocognitive Adverse Events (AEs) have been reported with all available Cystic Fibrosis Transmembrane conductance Regulator (CFTR) modulators in small groups of patients. We report our experience with a dose reduction strategy in 10 of our patients on elexacaftor/tezacaftor/ivacaftor (7.9% of total number of patients) who self-reported developing intense anxiety, irritability, sleep disturbance and/or mental slowness after initiation of full dose treatment. Standard dose elexacaftor/tezacaftor/ivacaftor resulted in 14.3 points improvement in mean Percent Predicted Forced Expiratory Volume in 1 s (ppFEV_1_), and a mean difference in sweat chloride of −39.3 mmol/L. We initially discontinued and/or reduced therapy according to the AEs severity, with a subsequent planned dose escalation every 4–6 weeks guided by sustainability of clinical effectiveness, absence of AEs recurrence, and patients’ preferences. Clinical parameters including lung function and sweat chloride were monitored for up to 12 weeks to assess ongoing clinical response to the reduced dose regimen. Dose reduction resulted in resolution of self-reported mental/psychological AEs, without loss of clinical effectiveness (ppFEV_1_ was 80.7% on standard dose, and 83.4% at 12 weeks on reduced dose; sweat chloride was 33.4 and 34 mmol/L on standard and reduced dose, respectively). Furthermore, in a subgroup of patients who completed 24 weeks of the reduced dose regimen, repeat low dose Computed Tomography imaging showed a significant response when compared to pre-initiation of elexacaftor/tezacaftor/ivacaftor.

## 1 Introduction

The prevalence of mental health disorders is high among people with Cystic Fibrosis (CF) (Quittner et al., 2014; Quittner et al., 2016). It is estimated that 5%–19% of CF adolescents and 13%–29% of CF adults have depression (Latchford and Duff, 2013; Quittner et al., 2014), along with 22% of CF adolescents and 32% of CF adults experiencing anxiety (Quittner et al., 2014). The psychological symptoms in CF have been linked to poor treatment adherence (Hilliard et al., 2015), worse clinical outcomes (Snell et al., 2014; Quittner et al., 2016), and greater healthcare utilization and costs (Snell et al., 2014).

Over the last decade, treatment with Cystic Fibrosis Transmembrane Conductance Regulator (CFTR) modulators has resulted in significant clinical benefits. More recently, a triple combination CFTR modulator using the next-generation corrector Elexacaftor in combination with Tezacaftor/Ivacaftor (ETI), has shown high clinical efficacy in patients homozygous or heterozygous for Phe508del mutation (Heijerman et al., 2019; Middleton et al., 2019; Barry et al., 2021). Despite the positive clinical outcomes for the majority of patients, mental health and neurocognitive Adverse Events (AEs) have been reported in real-world studies with all available CFTR modulators among small groups of patients (McKinzie et al., 2017; Talwalkar et al., 2017; Dagenais et al., 2020; Heo et al., 2022). The recently published Phase 3b clinical trial of ETI showed that one participant (1/87) in the ETI group discontinued treatment due to an adverse AE of anxiety and depression, and two participants (2/88) in the tezacaftor/ivacaftor group discontinued treatment due to AEs of psychotic disorder and obsessive-compulsive disorder (Sutharsan et al., 2022). In addition, a recently published real-world study showed an incidence of self-reported mental AEs in 7.1% of patients on ETI treatment (Spoletini et al., 2022).

The mechanism behind mental health AEs reported during treatment with CFTR modulators has not been fully illuminated, but the potential pathways include: 1) Drug-drug interaction between CFTR modulators and psychotropic medications through cytochrome P450 (Jordan et al., 2016; McKinzie et al., 2017). CFTR modulators, specifically ivacaftor and lumacaftor may affect the activities of cytochrome P450 isoenzymes (CYP2C9, CYPC19, CYP3A4), therefore this may alter the level of other cytochrome P450 substrates including selective serotonin reuptake inhibitors (SSRIs) and benzodiazepines. 2) The direct effect of CFTR modulators and their metabolites on the serotonin receptors and the CFTR receptors that are expressed ubiquitously in the nervous system (Marcorelles et al., 2014; Schneider et al., 2018).

The potential mental health AEs of CFTR modulator therapy, have focused our attention on the Phase 2 clinical trial (Keating et al., 2018). This trial demonstrated a variable but clinically significant response to different doses of the triple CFTR modulator ETI. Doses as low as 50 mg of VX-445 (elexacaftor) resulted in improvements in Percent Predicted Forced Expiratory Volume in 1 s (ppFEV_1_) and sweat chloride of almost 11% and −38 mmol/L respectively (Keating et al., 2018). In addition, the potential clinical effectiveness of a lower dose strategy based on this Phase 2 study, allowed us to develop an adaptive strategy where we adopted a dose reduction protocol in the subgroup of patients with self-reported intense anxiety, irritability, sleep disturbance and/or mental slowness, in an attempt to minimize AEs while continuing CFTR modulator therapy. We report our experience with a dose reduction strategy.

## 2 Case series description

### 2.1 Response to full standard dose of ETI

As a standard practice in our institution, along with standard clinical measures, all adult CF patients are screened for anxiety and depression prior to commencing ETI treatment (baseline) via a series of established questionnaires, specifically the Hospital Anxiety and Depression Scale (HADS), the Patient Health Questionnaire (PHQ-9), and General Anxiety Disorder-7 (GAD-7).

Between October 2020 and May 2021, we initiated ETI with 126 adult CF patients attending our service. Subsequently, a total of 10 patients (7.9% of patients on ETI) with no known psychological disorders or history of strong cytochrome P450 inducer or inhibitor use, developed self-reported anxiety, irritability, sleep disturbance and/or mental slowness within four weeks of initiation of full-dose treatment. None of these patients reported any anxiety or depression prior to ETI, and their baseline depression and anxiety screening questionnaires were within normal. The average weight and BMI of this group of patients at the start of ETI therapy were 68 kg (SD 13.9, min–max 48.7–86.4 kg) and 23.7 kg/m^2^ (SD 3.02, min–max 19.8–29.5 kg), respectively. All 10 patients were on a CFTR modulator therapy prior to switching to ETI (five of these patients were on ivacaftor, and five patients were on lumacaftor/ivacaftor). At the time of self-reported mental health issues, follow-up clinical parameters in this group of patients on full standard dose of treatment (as seen in Table 1) showed significant improvement in lung function (ppFEV_1_ was 14.3 points higher compared to baseline, *p* = 0.0134), and significant reduction in sweat chloride (mean of difference in sweat chloride was −39.3 mmol/L, *p* < 0.005), consistent with the findings of the clinical trials (Heijerman et al., 2019; Middleton et al., 2019; Barry et al., 2021). As a result, all patients wanted to stay on ETI treatment given the significant improvements they experienced with respiratory clinical parameters.

**TABLE 1:** Comparison of clinical efficacy parameters pretreatment, on full dose, and reduced dose of elexacaftor/tezacaftor/ivacaftor therapy.

	Genetic mutation	Pre- ETI treatment sweat chloride	Pre-ETI treatment FEV-1	Sweat chloride on full dose ETI	FEV-1 on full dose ETI	Sweat chloride at 4–6 weeks (on reduced dose ETI)	FEV-1 at 4–6 weeks (on reduced dose ETI)	Sweat chloride at 10–12 weeks (on reduced dose ETI)	FEV-1 at 10–12 weeks (on reduced dose ETI)	Outcome of self-reported mental AEs
Patient 1	Delta F508/Delta F508	95 mmol/L	3.8 L/79% predicted	39 mmol/L	4.27 L/107% predicted	46 mmol/L	4.20 L/106% predicted	46 mmol/L^a^	4.23 L/106% predicted^a^	Complete resolution
Patient 2	Delta F508/G551D	—	—	19 mmol/L	2.67 L/65% predicted	21 mmol/L	2.72 L/70% predicted	17 mmol/L^a^	2.7 L/69% predicted^a^	Complete resolution
Patient 3^b^	Delta F508/Delta F508	62 mmol/L	3.92 L/92% predicted	30 mmol/L	4.04 L/96% predicted	28 mmol/L	4.06 L/96% predicted	20 mmol//L^c^	4.14 L/98% predicted^c^	Complete resolution
Patient 4^b^	Delta F508/Delta F508	85 mmol/L	3.87 L/85% predicted	—	—	—	—	45 mmol/L^d^	4.49 L/98% predicted^d^	Complete resolution
Patient 5	Delta F508/G551D	51 mmol/L	2.73 L/69% predicted	37 mmol/L	3.78 L/95% predicted	29 mmol/L	3.98 L/101 % predicted	32 mmol/L^a^	4.26 L/108% predicted^a^	Complete resolution
Patient 6	Delta F508/Delta F508	74 mmol/L	3.36 L/84% predicted	56 mmol/L	3.32 L/83% predicted	41 mmol/L	3.40 L/85% predicted	51 mmol/L^a^	3.21 L/81% predicted^a^	Partial resolution
Patient 7	Delta F508/Delta F508	105 mmol/L	2.06 L/53% predicted	39 mmol/L	2.47 L/64% predicted	45 mmol/L	2.34 L/61% predicted	56 mmol/L^c^	2.35 L/61% predicted^c^	Complete resolution
Patient 8	Delta F508/Delta F508	89 mmol/L	1.3 L/48% predicted	19 mmol/L	1.83 L/68% predicted	—	—	15 mmol/L^c^	1.71 L/63% predicted^c^	Partial resolution
Patient 9	Delta F508/G551D	47 mmol/L	1.62 L/56% predicted	28 mmol/L	1.79 L/68% predicted	19 mmol/L^a^	1.70 L/65% predicted	24 mmol/L^a^	1.77 L/67% predicted^a^	Partial resolution

ETI, Elexacaftor/Tezacaftor/Ivacaftor; FEVI, Forced Expiratory Volume in 1 s; AEs, Adverse Events.

^a^ One tablet elexacaftor/tezacaftor/ivacaftor (100/50/75 mg) morning, and one tablet Ivacaftor 150 mg evening.

^b^ Initial complete discontinuation of ETI prior to dose reduction strategy.

^c^ Two tablets elexacaftor/tezacaftor/ivacaftor (200/100/150 mg) morning, (patients self-deviated from original plan).

^d^ One Tablet elexacaftor/tezacaftor/ivacaftor (100/50/75 mg) morning (Patient opted to remain on this dose given that he developed significant mental/psychological AEs within 2 weeks of standard-dose treatment initiation that necessitated hospital admission).

Patient 3: ETI therapy discontinued for 4 weeks, sweat chloride and ppFEV-1 were 99 mmol/L and 93%, respectively at the end of the washout period.

Patient 4: Patient opted to switch CFTR modulator therapy back to lumacaftor/ivacaftor, sweat chloride and ppFEV-1 while on lumacaftor/ivacaftor were 85 mmol/L and 91%, respectively. ETI therapy recommenced on reduced dose 4 months after initial experience.

This subgroup of patients (*n* = 10) was fully assessed by our multidisciplinary team. Patients who consented for further psychological assessment were referred to the appropriate service for psychological support and/or pharmacological therapy if indicated. Of those with reported mental health AEs, four patients were referred for psychological support, and required anxiolytic medications transiently that were discontinued within 2 weeks. None of the patients required long term psychotropics given resolution of AEs shortly after ETI discontinuation or dose reduction.

### 2.2 Dose reduction approach

**FIGURE 1:**
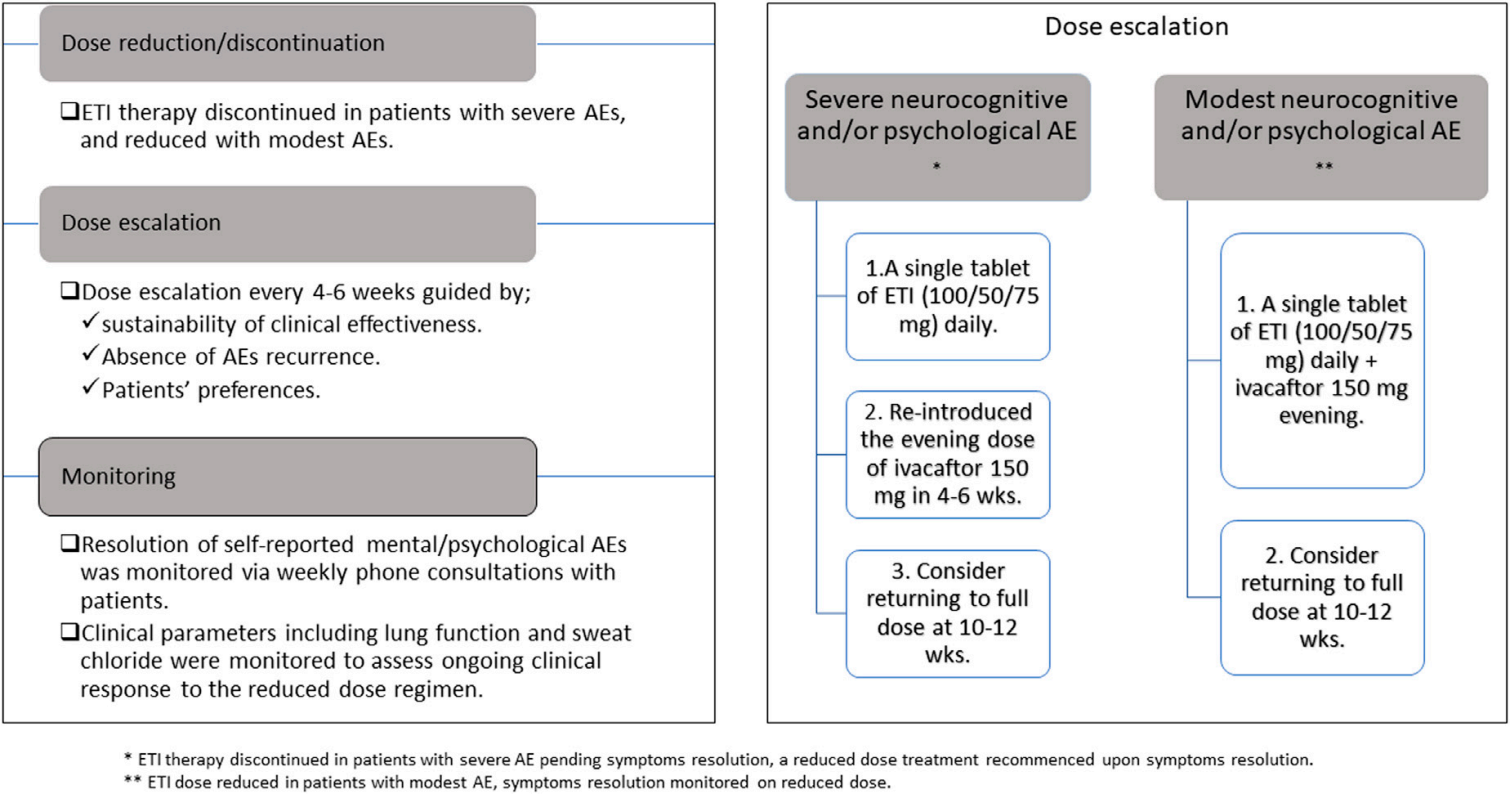
Outline of elexacaftor/tezacaftor/ivacaftor dose reduction approach.

We adopted a dose reduction approach (Figure 1) in an attempt to minimize self-reported AEs but sustain adequate CFTR modulation. Our approach to dose reduction was developed in consultation with the CF multidisciplinary team and was as follows:

• In patients with severe self-reported neurocognitive and/or psychological AEs, we discontinued therapy pending AEs resolution. We subsequently re-introduced therapy at a lower dose, starting with a single tablet of ETI (100/50/75 mg) daily. We planned to reintroduce the evening dose of ivacaftor 150 mg at 4–6 weeks and considered returning to full dose therapy at 10–12 weeks. Dose escalation was guided by ongoing monitoring of clinical efficacy parameters, absence of psychological/neurocognitive AEs and patients’ preferences.
• In patients with modest self-reported AEs, we reduced CFTR modulator therapy dose to a single tablet of ETI (100/50/75 mg) daily and continued the evening dose of ivacaftor 150 mg. We considered returning to full dose therapy at 10–12 weeks.

To assess ongoing clinical response to the reduced dose regimen, clinical efficacy parameters including lung function (clinical marker of sustained response), and sweat chloride (indirect measure of CFTR function restoration) (Mall et al., 2020) were recorded every 4–6 weeks for the first 12 weeks on reduced dose therapy. Members of the CF multidisciplinary team (CF clinical nurse specialist) contacted these patients weekly via phone to monitor any changes in self-reported mental/psychological AEs. The assessment of AEs resolution was subjective based on patients’ perception of symptoms.

### 2.3 Response to reduced dose regimen

**FIGURE 2:**
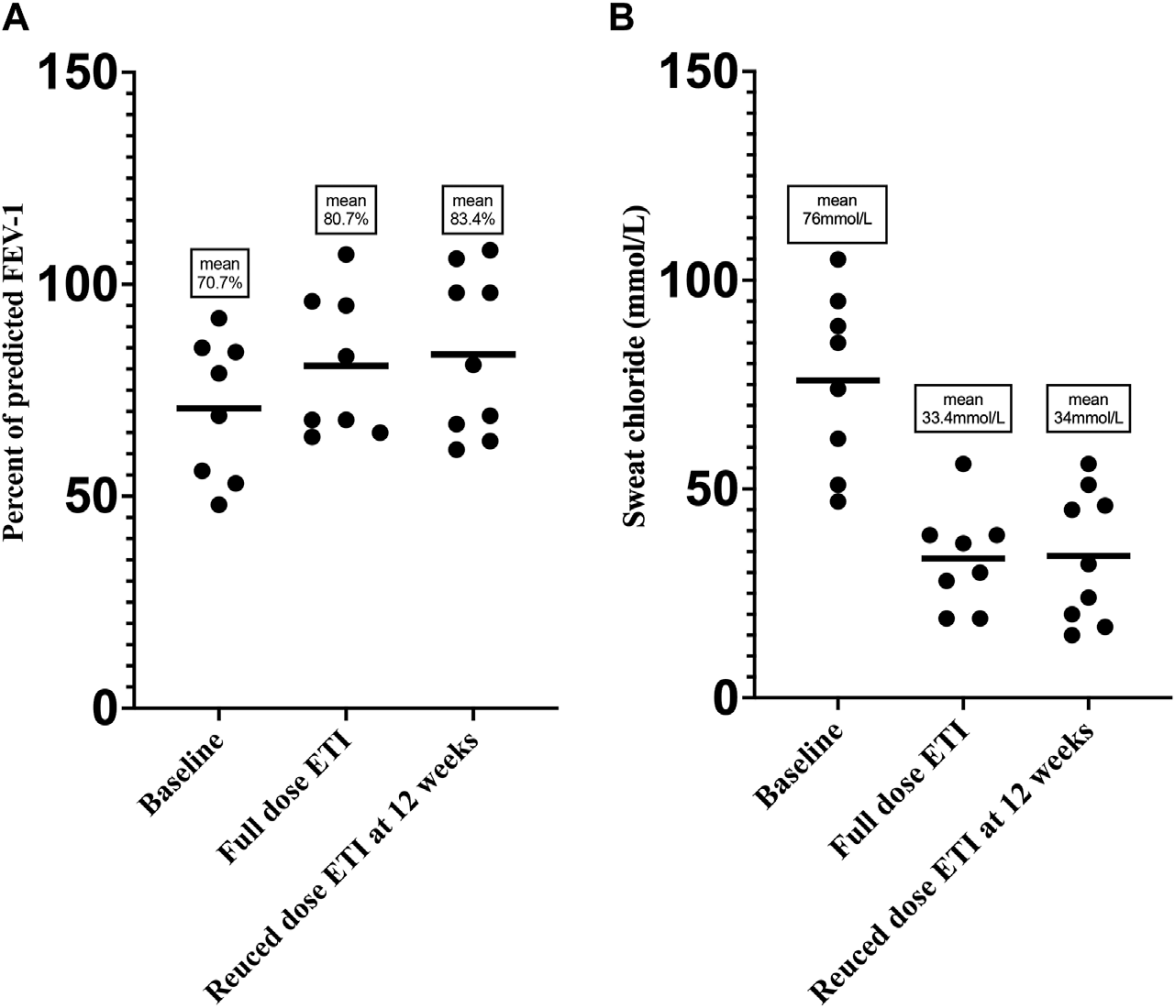
Change in **(A)** FEV_1_ and **(B)** sweat chloride at baseline, on full standard dose, and reduced dose of ETI therapy.

A total of nine patients commenced the dose reduction protocol and the remaining one patient opted to discontinue ETI and switched back to ivacaftor. Table 1 and Figure 2 demonstrates individual patient responses and dosing. Follow-up data showed resolution of self-reported mental/psychological AEs within 2 weeks in most patients, while their clinical efficacy parameters at 4–6 and 10–12 weeks on reduced dose were comparable to those on original full dose (mean ppFEV_1_ was 80.7% on standard dose ETI, compared to 83.4% at 12 weeks on reduced dose; mean sweat chloride was 33.4 and 34 mmol/L on standard and at 12 weeks on reduced dose, respectively).

**FIGURE 3:**
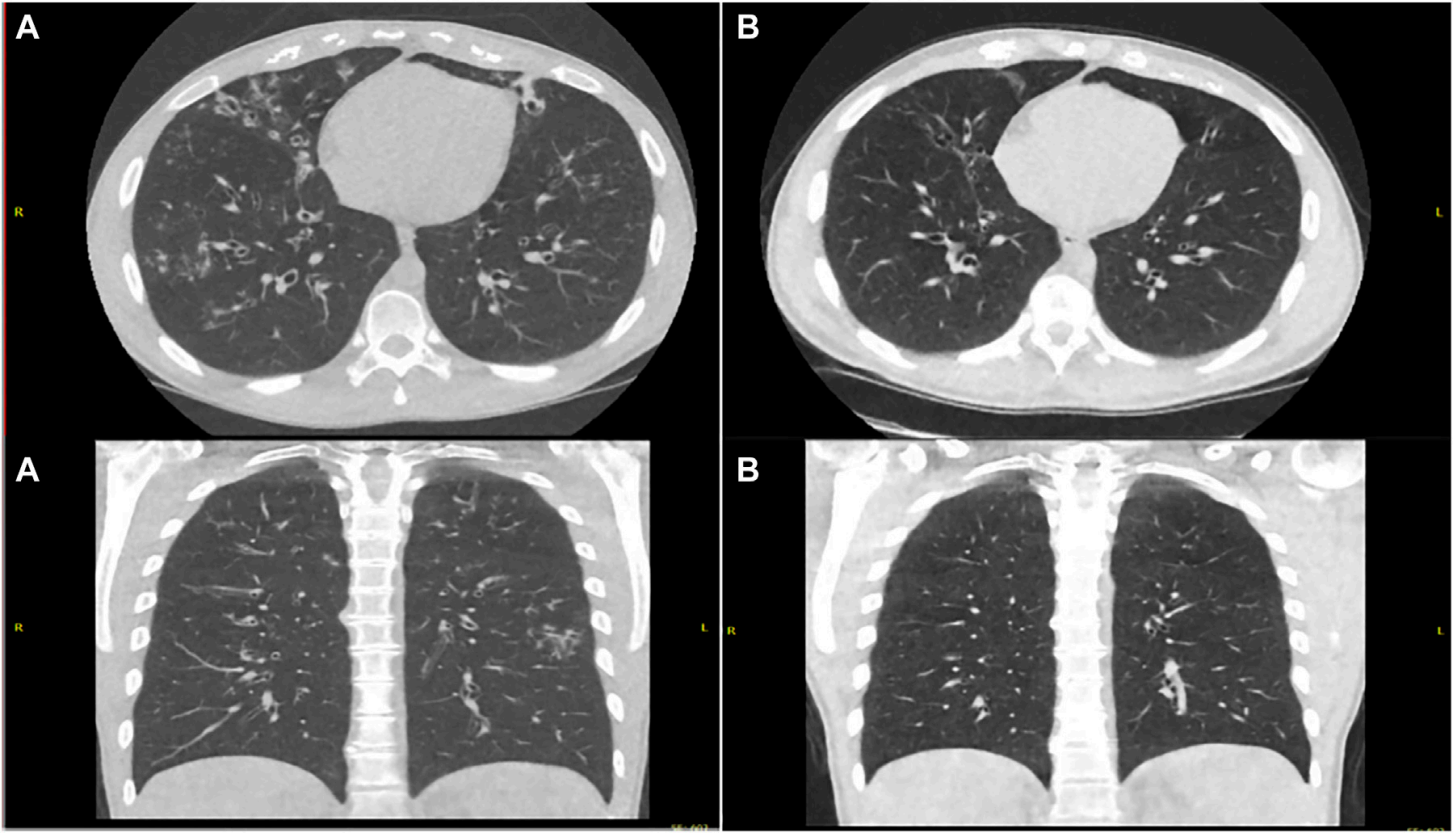
Ultra-Low Dose CT Thorax pretreatment **(A)** and post modified dose regimen **(B)** showing reduced burden of bronchiectasis, reduced caliber of bronchiectatic airways, decreased bronchial wall thickening and resolution of tree in bud opacification changes.

**FIGURE 4:**
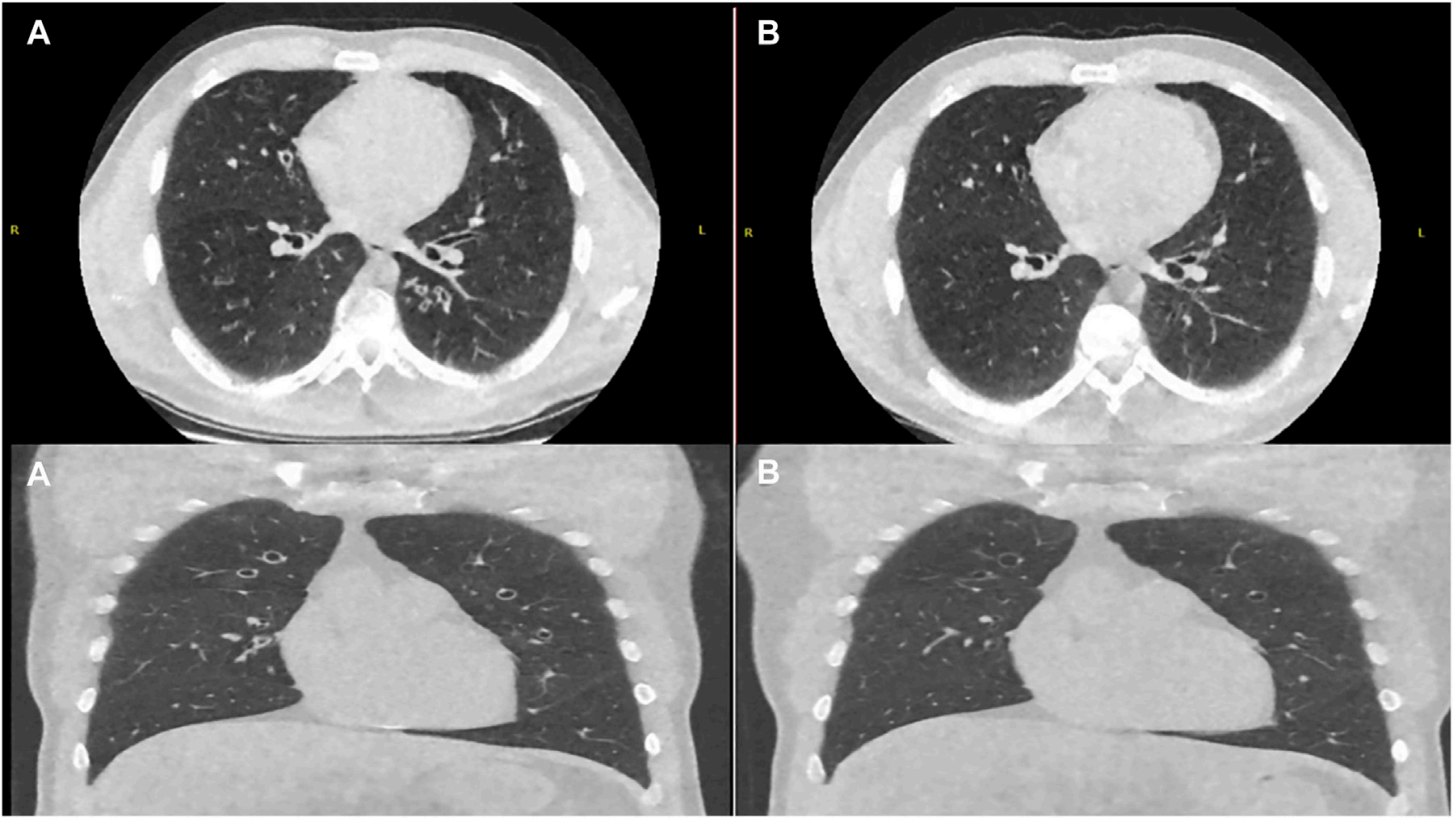
Axial and Coronal Ultra-Low Dose CT Thorax pretreatment **(A)** and post modified dose regimen **(B)** showing reduction in the degree of bronchial wall thickening and mucoid impaction.

Whilst all nine patients adopted a sustained dosed reduction strategy, we acknowledge that two patients self-deviated from our proposed approach as highlighted in Table 1. At 12 weeks of the reduced dose regimen, six patients elected to remain on a reduced dose regimen and three patients switched back to full dose treatment. Repeat imaging in patients who completed 24 weeks on reduced dose regimen at the time of preparation of this manuscript (*n* = 2 of six patients) showed a significant improvement compared to imaging before initiation of modulator therapy (Figures 3, 4).

## 3 Discussion

Over a 12 weeks period, dose reduction in our cohort of patients who developed self-reported mental health AEs resulted in the resolution of AEs without significant change to the clinical response achieved while on full dose of therapy. In addition, two of these patients had radiological improvement at 24 weeks on this regimen. That said, the long-term outcomes of reduced dose of ETI therapy remain unclear. Continued close real-world monitoring of this group is critical and ongoing.

We believe the mental health AEs in our cohort may be related to the add on effect of the CFTR correctors elexacaftor and/or tezacaftor, as all patients in this subgroup were previously on either ivacaftor or lumacaftor/ivacaftor prior to ETI therapy. One of the hypothesized mechanisms behind the mental health AEs of ETI therapy is drug-drug interaction between ETI and psychotropic medications, but none of the patients in this group were on any psychotropic medications, this would suggest that the mental health AEs in our cohort are likely directly related to ETI therapy. Furthermore, the mental health AEs resolved shortly after dose reduction or discontinuation of ETI therapy. A recently published case series outlining the real-world experience of a UK CF centre not only demonstrated a similar experience with the self-reporting of mental health AEs, but also a similar incidence of 7.1% (Spoletini et al., 2022). This further supports our belief that these events may be related to ETI therapy. Moreover, the UK case series also exemplifies that dose adjustment of ETI can improve mental health AEs while sustaining clinical effectiveness, which further validates our finding and approach (Spoletini et al., 2022). Currently, there is no standardised dose reduction strategy, we attempted to achieve this but even in our case series patients deviated in their approach to dose reduction. Moving forward, as we understand more about this possible AEs a standardised strategy would be helpful to the clinical community.

Based on recent real-world analysis of serum levels of ETI in 78 adult CF patients during routine outpatient visits, 41.1% (*n* = 37) of patients had elevated serum levels of elexacaftor compared to known Pharmacokinetic (PK) values of elexacaftor (Naehrig et al., 2022). This raises the question: are we reducing the dose of ETI in our cohort of patients with self-reported mental health AEs, or are we just simply optimizing the treatment dose? The access to routine drug levels for CFTR modulators remains an issue for clinical services and access to these levels could answer this question. In addition, it could allow for further optimization of dose reduction strategies for patients with self-reported AEs.

The limitations of this work is that it reflects a single centre experience, with a modest number of cases reported. The roll out of ETI therapy was during the COVID-19 pandemic, which enforced social restrictions and isolation on vulnerable populations, such as CF patients. This in addition to concerns regarding the effect of COVID-19 on CF patients health, may have contributed to self-reported anxiety among our patients. That said, one could also hypothesise that the close monitoring of these patients after ETI dose reduction at a time where a social restriction measure was imposed on them, may have had a placebo effect in reassuring some of these patients and contributed to the improvements seen in their mental health status post ETI dose modification. However, the temporal relationship between drug initiation and AEs makes us believe that this happened independently of the pandemic. Also, the rapid and significant change in the wellbeing of CF patients post CFTR modulator therapy, may require patients to make changes to lifestyle and future planning which could potentially contribute to the self-reported anxiety.

Our real-world data and published work to date highlight that some patients do not tolerate standard full dose of CFTR modulator therapy. There is a need in small groups of patients who develop AEs, to individualize dosage so as to minimize AEs whilst continuing CFTR modulator therapy. Access to routine drug levels for ETI will compliment clinical/sweat chloride monitoring and guide dose adjustment. Routine drug levels for ETI are not made routinely available to the clinical CF community, which may potentially prohibit CF clinicians from prescribing lower dose regimens to patients who develop neuropsychiatric and/or neurocognitive AEs, for instance. Increased awareness and reporting of real-world adverse events from CFTR modulators is critical.

## Data Availability

The raw data supporting the conclusion of this article will be made available by the authors on reasonable request, without undue reservation.
